# Antiproliferative Activity of *Cinnamomum cassia* Constituents and Effects of Pifithrin-Alpha on Their Apoptotic Signaling Pathways in Hep G2 Cells

**DOI:** 10.1093/ecam/nep220

**Published:** 2011-05-02

**Authors:** Lean-Teik Ng, Shu-Jing Wu

**Affiliations:** ^1^Department of Agricultural Chemistry, National Taiwan University, No.1, Roosevelt Road Section 4, Taipei 106, Taiwan; ^2^Department of Nutritional Health, Chia-Nan University of Pharmacy and Science, 60, Erh-Jen Road, Section 1, Jen-Te, Tainan 717, Taiwan

## Abstract

Cinnamaldehyde (Cin), cinnamic acid (Ca) and cinnamyl alcohol (Cal), major constituents of *Cinnamomum cassia*, have been shown to possess antioxidant, anti-inflammatory, anticancer and other activities. In this study, our aim was to evaluate the antiproliferative activity of these compounds in human hepatoma Hep G2 cells and examine the effects of pifithrin-alpha (PFT**α**; a specific p53 inhibitor) on their apoptotic signaling transduction mechanism. The antiproliferative activity was measured by XTT assay. Expression of apoptosis-related proteins was detected by western blotting. Results showed that at a concentration of 30 **μ**M, the order of antiproliferative activity in Hep G2 cells was Cin > Ca > Cal. Cin (IC_50_ 9.76 ± 0.67 **μ**M) demonstrated an antiproliferative potency as good as 5-fluorouracil (an anti-cancer drug; IC_50_ 9.57 ± 0.61 **μ**M). Further studies on apoptotic mechanisms of Cin showed that it downregulated the expression of Bcl-_XL_, upregulated CD95 (APO-1), p53 and Bax proteins, as well as cleaving the poly (ADP-ribose) polymerase (PARP) in a time-dependent pattern. PFT**α** pre-incubation significantly diminished the effect of Cin-induced apoptosis. It markedly upregulated the anti-apoptotic (Bcl-_XL_) expression and downregulated the pro-apoptotic (Bax) expression, as well as effectively blocking the CD95 (APO-1) and p53 expression, and PARP cleavage in Cin-treated cells. This study indicates that Cin was the most potent antiproliferative constituent of *C. cassia*, and its apoptotic mechanism in Hep G2 cells could be mediated through the p53 induction and CD95 (APO-1) signaling pathways.

## 1. Introduction

*Cinnamomum cassia* Presl. (Lauraceae) has been traditionally used to treat dyspepsia, gastritis, blood circulation disturbances and inflammatory diseases [1]; it is an important ingredient in herbal preparations [2, 3]. Its major components cinnamaldehyde (Cin), cinnamic acid (Ca) and cinnamyl alcohol (Cal) were reported to have various biological activities. For example, Ca possessed antioxidant, anti-inflammatory and anticancer properties [4–6]. Cal is a fragrance ingredient, which is used in cosmetics, shampoos, soaps and other toiletries [7]. Cin exhibited antifungal, antipyretic, antioxidant, antimicrobial and larvicidal activities [8–10], as well as modulating T-cell differentiation [11]. In anticancer study, Cin was active against human liver, lung and leukemia cancer cells [12–14]. However, the antihepatoma activity and mechanism(s) of action of Cin, Ca and Cal in Hep G2 cells have never been investigated.

In our previous studies, p53 induction and MAPK pathways were shown to require for Cin-mediated apoptosis in PLC/PRF/5 cells [14]. In this study, we examined the anti-hepatoma activity of *C. cassia* bioactive components in Hep G2 cell lines. Hep G2 cells are highly differentiated cells [15], whereas PLC/PRF/5 is less divided but highly migrated hepatoma cells [16]. It functionally behaves as highly differentiated liver parenchymal cells and is karyologically distinguishable from PLC/PRF/5 due to the presence of trisomy 6 (pter leads to q14) and a long arm of chromosome 15q+ [15]. Hep G2 not PLC/PRF/5 cells secrete IGF carrier protein [17] and produce *α*-2-plasmin inhibitor (*α*-PI), a physiological inhibitor [18]. PLC/PRF/5 cells possess mutant or null p53 protein whereas Hep G2 cells have wild-type p53 protein [14, 19].

Apoptosis is a process in which cell death is initiated and completed in an orderly manner through activation and/or synthesis of gene products necessary for cell destruction [20]. p53 directly activated the promoter of the CD95 (APO-1) gene in response to DNA damage by anticancer agents. The upregulation of the CD95 (APO-1) death receptor was only observed in cells with wild-type p53, but not in cells with mutant or null p53 [19]. Activation of p53 (a tumor suppressor protein) is known to result in the altered transcription of a wide variety of genes involving in-cell metabolism, cell cycle regulation and apoptosis [21, 22]. Both pro-apoptotic (Bax, Bak, Bid, Noxa, etc.) and anti-apoptotic (Bcl-2, Bcl-_XL_, Mcl-1, Bcl-w, etc.) proteins have been reported to be key regulators of apoptosis [23]. Genes transcriptionally upregulated by p53 are implicated in promoting apoptosis, which includes the Bcl-2 family members (e.g., Bax, Bak) and Noxa gene proteins [24–26]. The p53-dependent apoptotic pathway can lead to the cellular protein cleavage (e.g., PARP), DNA damage and cell death.

Pifithrin-alpha (PFT*α*; a p53 inhibitor) is able to suppress p53-mediated transactivation [27]. It significantly decreased p53 expression on wild type p53 cells, but had no effect on mutant p53 cells or p53-deficient cells [28]. In this study, our aims were (i) to evaluate the antiproliferative activity of Cin, Ca and Cal in human hepatoma Hep G2 (CD95-positive) cells; (ii) to investigate the role of p53, Bcl-2 family proteins (Bax and Bcl-_XL_) and PARP in Cin-mediated apoptosis; and (iii) to study the effects of PFT*α* on p53 and Bcl-2 family proteins, as well as PARP cleavage in Hep G2 cells.

## 2. Methods

### 2.1. Chemicals

Cin, Ca and Cal with purity greater than 98% were purchased from Merck Chemical Industries (Germany). Pifithrin-alpha (PFT*α*) was purchased from Calibiochem (San Diego, CA, USA). Dulbecco's modified Eagle's medium (DMEM), dimethyl sulfoxide (DMSO), penicillin, streptomycin, aprotonin, trypsin-EDTA, sodium 3,3′-[1-(phenylaminocarbonyl)-3,4-tetrazolium]-*bis*(4-methoxy-6-nitro) benzene sulfonic acid (XTT), 5-flurouracil (5FU) and anti-*β*-actin body were purchased from Sigma Chemical Co. (St. Louis, MO, USA). Fetal bovine serum (FBS) was obtained from GIBCO BRL (Gaithersburg, MD, USA). The anti-Bax, anti-Bcl-_XL_, anti-CD95 (APO-1/CD95), anti-p53, anti-PARP, anti-rabbit IgG and anti-mouse IgG bodies were purchased from PharMingen (San Diego, CA, USA).

### 2.2. Preparation of Cell Culture and Test Solutions

The human hepatoma Hep G2 cells (ATCC HB-8065) were obtained from the American Type Culture Collection (Rockville, MD, USA). They were grown in DMEM supplemented with 10% FBS, 100 units mL^−1^ penicillin and 100 *μ*g mL^−1^ streptomycin at 37°C in a humidified atmosphere of 5% CO_2_. All stock solutions were prepared in DMSO at a concentration of 10 mM and stored at –20°C until use. The concentrations of test compounds used for this study were 1, 10, 30, 50 and 70 *μ*M, which were freshly prepared for each experiment with a final DMSO concentration of 0.1%. Control samples were always treated with the same amount of DMSO (0.1% v/v) as used in the corresponding experiments.

### 2.3. Analysis of Antiproliferative Activity

Cells were seeded at a density of 1×10^5^ cells per well on 12-well plates. They were then treated with various concentrations of Cin, Ca, Cal or 0.1% DMSO (control) for 0, 6, 12 and 24 h. After treatments, cells were washed once before adding 100 *μ*L of FBS-free medium containing XTT, followed by incubating at 37°C for 4 h. The absorbance of samples was measured with an ELISA reader (Bio-Rad, USA) at a test wavelength of 492 nm and a reference wavelength of 690 nm.

### 2.4. Pifithrin-alpha (PFT*α*; A p53 Inhibitor) Treatment

Cells were assured to grow normally before inhibitor treatment. Confluent cells in each experiment were pretreated for 1 h with 30 *μ*M PFT*α* dissolving in DMSO. The cells were treated with 30 *μ*M Cin or 0.1% DMSO (control) for 24 h and then harvested for apoptotic assays. The inhibitor was prepared according to the manufacturer's instructions. In all experiments, the vehicle used to prepare stock solutions was noted to have no effect on the cell viability.

### 2.5. Analysis of PFT*α* Effects on Cin-Induced Apoptosis

Cells were seeded at a density of 1×10^5^ cells per well onto 12-well plates. They were treated with 0.1% DMSO (control) or 30 *μ*M Cin only or pretreated with 30 *μ*M PFT*α* for 1 h before adding 30 *μ*M Cin. After 24 h of treatment, cells were washed once before adding 100 *μ*L of FBS-free medium containing XTT. After 4 h of incubation at 37°C, the absorbance of samples was measured with an ELISA reader.

## 3. Western Immunoblot Analysis

Cells were harvested and subjected to procedures described previously [14]. The specific primary antibodies used in this study were anti-Bax (1 : 250), anti-Bcl-_XL_ (1 : 500), anti-CD95/APO-1 (1 : 5000), anti-p53 (1 : 500) and anti-PARP (1 : 500) antibodies.

### 3.1. Statistical Analysis

Values were evaluated by one-way ANOVA, followed by Duncan's multiple range tests using the Statistical Analysis System (SAS Institute, Cary, NC, USA). Differences were considered significant when the *P*-value was < .05.

## 4. Results

### 4.1. Antiproliferative Activity of Cin, Ca and Cal in Hep G2 Cells

**Figure 1:**
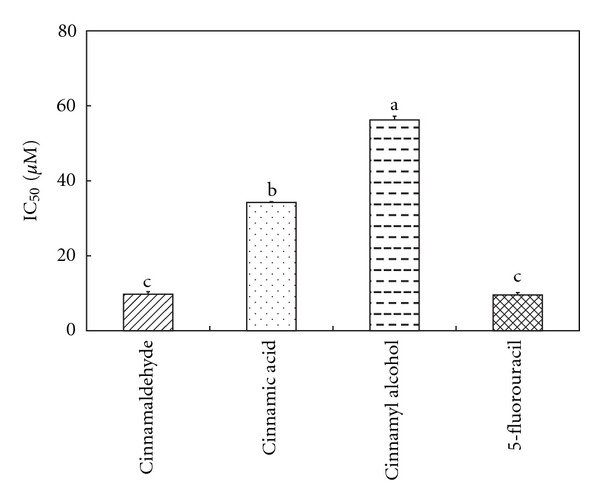
IC_50_ values of antiproliferative activity of cinnamaldehyde, cinnamic acid and cinnamyl alcohol. The data shown are means ± SD of three independent experiments. Bars with different alphabetical letters were significantly different at *P* < .05.

To examine the antiproliferative effect of Cin, Ca and Cal on Hep G2 cells, cells were treated with different concentrations of these compounds using XTT assay. Results showed that all test compounds exhibited inhibitory effects on the growth of Hep G2 cells, with an IC_50_ value of 9.76 ± 0.67 *μ*M for Cin, 34.20 ± 0.99 *μ*M for Ca and 58.30 ± 1.49 *μ*M for Cal (Figure 1). The potency of Cin was closed to that of the positive control 5 FU (IC_50_ 9.57 ± 0.61 *μ*M). Hence, it was selected for detailed study of apoptotic mechanism.

### 4.2. Inhibition of Cell Proliferation by Cin

As shown in Table 1, Cin inhibited the proliferation of Hep G2 cells in a dose- and time-dependent manner. Compared with the control, Cin at 30 *μ*M caused a nearly 71% inhibition of cell growth as demonstrated by a significant increase in the number of apoptotic cells. This concentration of Cin was used in all further experiments.

### 4.3. CD95 (APO-1/CD95) Is Involved in Cin-Induced Apoptosis

**Figure 2:**
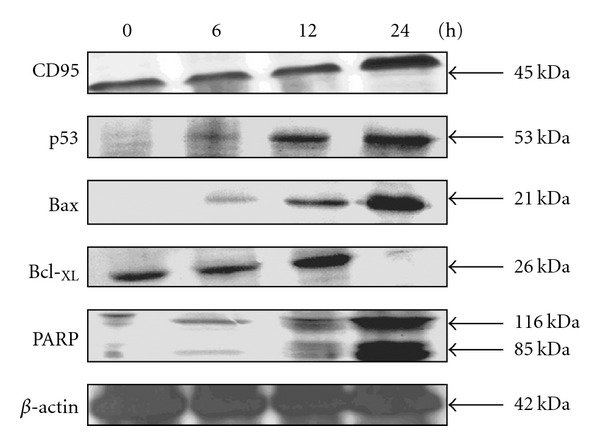
Effects of apoptotic signal transduction factors (CD95, p53, Bax, Bcl-_XL_ and PARP) in Cin-induced cell death. Cells were treated with 30 *μ*M Cin for 0, 6, 12 and 24 h and then harvested for Western blotting analysis. *β*-Actin was used as a positive control.

CD95 (APO-1/CD95) pathway has been well documented to participate in certain anticancer drugs-induced apoptosis. In this study, results showed that Cin caused a time-dependent increase in the CD95 (APO-1/CD95) protein expression in Hep G2 cells (Figure 2).

### 4.4. Cin Treatment Downregulates Bcl-_XL_ and Upregulates Bax and p53 Protein Levels

Treatment with 30 *μ*M Cin resulted in the downregulation of the anti-apoptotic (Bcl-_XL_) and the upregulation of the pro-apoptotic (Bax) proteins in a time-dependent fashion (Figure 2). The expression of Bcl-_XL_ protein was noted to disappear after 24 h of Cin treatment. As expected, Cin did cause an increase in the level of p53 as Hep G2 cells contain wild-type p53.

### 4.5. Cin-Induced Apoptosis Exhibits PARP Cleavage

To further confirm the Cin-induced apoptosis, cells were treated with Cin for 0, 6, 12 and 24 h. PARP cleavage was determined by immunoblotting analysis. Results showed that PARP proform (molecular mass, 116 kDa) was cleaved to give an 85 kDa fragment in Cin-treated cells at 12 and 24 h after treatment (Figure 2).

### 4.6. Pifithrin *α* (PFT*α*) Prevents the Cin-Induced Apoptosis

**Figure 3:**
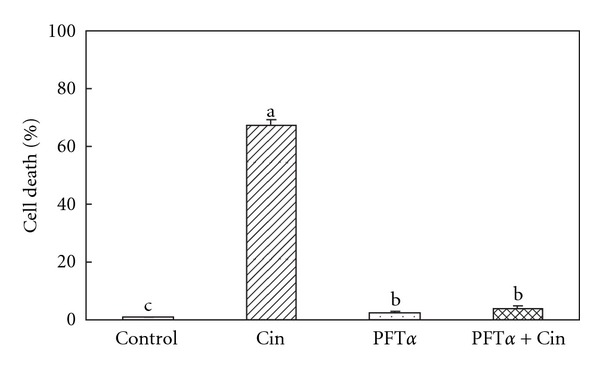
Effects of the p53 inhibitor (PFT*α*) on Cin-induced cell death. The data represent means ± SD of three independent experiments. Bars with different alphabetical letters were significantly different at *P* < .05.

To determine whether the Cin induction of apoptosis was affected by the presence of 30 *μ*M PFT*α*, Hep G2 cells were pre-incubated with PFT*α* for 1 h, and then induced to undergo apoptosis by treatment with Cin. Results showed that PFT*α* significantly (*P* < .05) inhibited the Cin-induced Hep G2 cell death (Figure 3).

### 4.7. PFT*α* Suppresses the Expression of CD95 (APO-1/CD95), p53 and Bax Proteins

**Figure 4:**
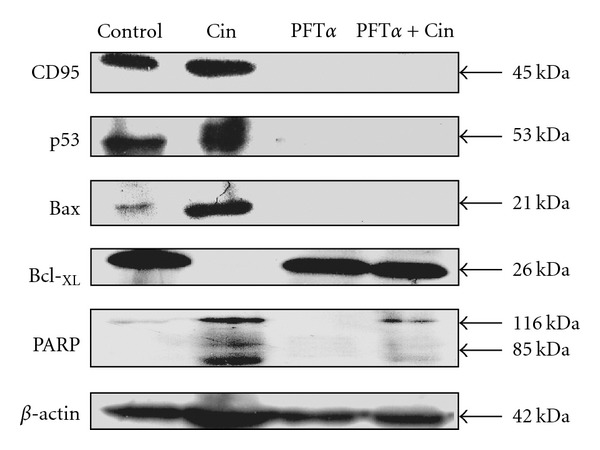
Effects of p53 inhibitor (PFT*α*) on Cin-induced apoptosis. Hep G2 cells were treated without or with 30 *μ*M PFT*α* for 1 h, and then in the presence or absence of 30 *μ*M Cin for 24 h. The total cell lysate was then analyzed by western blotting analysis. *β*-Actin was used as a positive control.

To evaluate the relative role of CD95 (APO-1/CD95), p53, Bcl-2 family (Bax, Bcl-_XL_) proteins and PARP cleavage in the Cin-induced apoptotic events, cells were pretreated with PFT*α*. Results displayed that pre-incubation the Hep G2 cells with 30 *μ*M PFT*α* alone and 30 *μ*M PFT*α* + 30 *μ*M Cin effectively inhibited the expression of Bax, p53 and CD95, as well as the cleavage of PARP (Figure 4). These pretreatment also prevented the downregulation of Bcl-_XL_ in cells.

## 5. Discussion

We have demonstrated that the major components of *C. cassia*, that is, Cin, Ca and Cal possessed a different magnitude of antiproliferative effect on human hepatoma Hep G2 cells. Among them, Cin was the most potent compound, which exhibited an IC_50_ value closed to that of the commercial anticancer agent 5 FU.

Cin has been shown to possess antitumor activity through inhibiting cell proliferation and inducing cell apoptosis [29–31]. Its inhibitory effect on cell cycle progression was demonstrated to be through the arrest of the S phase in human PLC/PRF/5 cells [31]. In this study, the effect of Cin on Hep G2 cell apoptosis was noted to be on the CD95 (APO-1/CD95) signal transduction and p53 pathways. It was also found that pretreatment with a p53 inhibitor (PFT*α*) could block the process of programmed cell death and prevent the apoptotic signal transduction pathway.

Several studies have shown that the Bcl-2 family of proteins is the central of apoptotic regulation [32, 33]. Overexpression of Bcl-2 and Bcl-_XL_ aborts the apoptotic response while Bax, Bid and Bak activity promotes cell death [32]. Our results displayed that Cin activated wild-type p53 and caused an up-expression of Bax as well as triggering the down-expression of Bcl-_XL_ with a subsequent promotion of the apoptotic activity in Hep G2 cells. p53 has been reported to mediate Bax upregulation [25]. It is possible that the Cin-mediated activation of Bax triggers the cleavage of PARP and lead to a p53-dependent pathway. The apoptotic induction in wild-type p53 cells has also been noted on taiwanin A and gambogic acid [34, 35].

**Figure 5:**
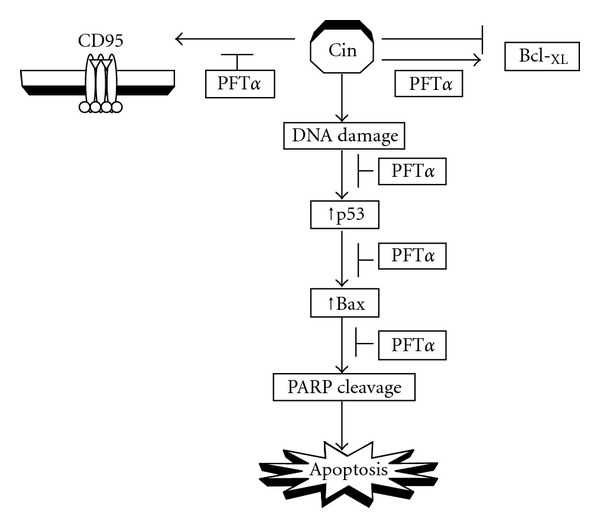
The mode of action of PFT*α* on the Cin-mediated apoptosis in Hep G2 cells.

PFT*α*, a small molecule identified as an inhibitor of p53 transcriptional activity, has been shown to protect against the toxic side effects of anticancer treatment to the normal tissues [36, 37]. It may also interfere with the apoptosis of tumor cells that sense DNA damage in response to genotoxic stress [38]. In this study, PFT*α* was able to completely inhibit the modulation of CD95 (APO-1/CD95), p53 and Bax proteins, and the suppression of the PARP cleavage in Cin-treated cells. This indicates that PFT*α* significantly prevented Cin-mediated apoptosis through blocking the expression of apoptotic signal factors such as CD95 (APO-1/CD95), Bax, p53 and PARP degradation in Hep G2 cells (Figure 5).

## 6. Conclusion

Among the major components of *C. cassia*, Cin has demonstrated to possess the most potent antiproliferative activity in Hep G2 cells. It was the first study to demonstrate the role of CD95 (APO-1/CD95) and p53 in the Cin-induced apoptotic signaling. In addition, PFT*α* was found to markedly block the Cin-induced apoptosis through upregulating the anti-apoptotic (Bcl-_XL_) and downregulating the pro-apoptotic (Bax) proteins, as well as suppressing the PARP cleavage. Importantly, PFT*α* completely attenuated the activation of CD95 (APO-1/CD95) and p53 in Cin-treated cells. These results suggest that the modulation of apoptotic pathways through the CD95 (APO-1/CD95), p53, PARP cleavage and Bcl-2 family proteins signaling transductions could be an important therapeutic goal in the prevention and treatment of cancer.

## Figures and Tables

**Table 1:** Effects of Cin-induced apoptosis on human Hep G2 cells.

Cin treatment (*μ*M)	Apoptotic cells (%)			
	0 h	6 h	12 h	24 h
0	0.10 ± 0.00^c^	0.10 ± 0.08^d^	0.10 ± 0.08^d^	0.13 ± 0.05^d^
1	0.50 ± 0.16^c^	8.25 ± 0.94^c^	17.07 ± 0.74^c^	41.13 ± 0.74^c^
10	5.00 ± 0.82^b^	13.20 ± 0.80^b^	45.96 ± 0.23^b^	50.10 ± 0.70^b^
30	16.98 ± 0.14^a^	38.67 ± 2.49^a^	60.32 ± 0.50^a^	70.73 ± 3.03^a^

After treatment, cell numbers were estimated by the XTT assay. Data are presented as means ± SD of three independent experiments. Values within the column with different superscript letters were significant at *P* < .05.

## References

[1] Lou ZQ, Qin B (1995). *Species Systematization and Quality Evaluation of Commonly Used Chinese Traditional Drugs*.

[2] Shijie Z, Moriya J, Yamakawa J, Chen R, Takahashi T, Sumino H (2008). Mao-to prolongs the survival of and reduces TNF-*α* expression in mice with viral myocarditis. *Evidence-Based Complementary and Alternative Medicine*.

[3] Chen FP, Jong MS, Chen YC, Kung YY, Chen TJ, Chen FJ (2009). Prescriptions of Chinese herbal medicines for insomnia in Taiwan during 2002. *Evidence-Based Complementary and Alternative Medicine*.

[4] Zhang LP, Ji ZZ (1992). Synthesis, antiinflammatory and anticancer activity of cinnamic acids, their derivatives and analogues. *Yao Xue Xue Bao*.

[5] Akao Y, Maruyama H, Matsumoto K (2003). Cell growth inhibitory effect of cinnamic acid derivatives from propolis on human tumor cell lines. *Biological and Pharmaceutical Bulletin*.

[6] Foti MC, Daquino C, Geraci C (2004). Electron-transfer reaction of cinnamic acids and their methyl esters with the DPPH^•^ radical in alcoholic solutions. *The Journal of Organic Chemistry*.

[7] Letizia CS, Cocchiara J, Lalko J, Lapczynski A, Api AM (2005). Fragrance material review on cinnamyl alcohol. *Food and Chemical Toxicology*.

[8] Lin C-C, Wu S-J, Chang C-H, Ng L-T (2003). Antioxidant activity of Cinnamomum cassia. *Phytotherapy Research*.

[9] Cheng SS, Liu JY, Tsai KH, Chen WJ, Chang ST (2004). Chemical composition and mosquito larvicidal activity of essential oils from leaves of different *Cinnmomum osmophloeum* provenances. *Journal of Agricultural and Food Chemistry*.

[10] Kim H-O, Park S-W, Park H-D (2004). Inactivation of *Escherichia coli* O157:H7 by cinnamic aldehyde purified from Cinnamomum cassia shoot. *Food Microbiology*.

[11] Koh WS, Yoon SY, Kwon BM, Jeong TC, Nam KS, Han MY (1998). Cinnamaldehyde inhibits lymphocyte proliferation and modulates T-cell differentiation. *International Journal of Immunopharmacology*.

[12] Moon KH, Pack MY (1983). Cytotoxicity of cinnamic aldehyde on leukemia L1210 cells. *Drug and Chemical Toxicology*.

[13] Imai T, Yasuhara K, Tamura T (2002). Inhibitory effects of cinnamaldehyde on 4-(methylnitrosamino)-1-(3-pyridyl)-1-butanone-induced lung carcinogenesis in rasH2 mice. *Cancer Letters*.

[14] Wu S-J, Ng L-T (2007). MAPK inhibitors and pifithrin-alpha block cinnamaldehyde-induced apoptosis in human PLC/PRF/5 cells. *Food and Chemical Toxicology*.

[15] Simon D, Aden DP, Knowles BB (1982). Chromosomes of human hepatoma cell lines. *International Journal of Cancer*.

[16] Zvibel I, Halay E, Reid LM (1991). Heparin and hormonal regulation of mRNA synthesis and abundance of autocrine growth factors: relevance to clonal growth of tumors. *Molecular and Cellular Biology*.

[17] Moses AC, Freinkel AJ, Knowles BB, Aden DP (1983). Demonstration that a human hepatoma cell line produces a specific insulin-like growth factor carrier protein. *Journal of Clinical Endocrinology and Metabolism*.

[18] Saito H, Goodnough LT, Knowles BB, Aden DP (1982). Synthesis and secretion of alpha 2-plasmin inhibitor by established human liver cell lines. *Proceedings of the National Academy of Sciences of the United States of America*.

[19] Muller M, Wilder S, Bannasch D (1998). p53 activates the CD95 (APO-1/Fas) gene in response to DNA damage by anticancer drugs. *Journal of Experimental Medicine*.

[20] Huang J, Wu L, Tashiro SI, Onodera S, Ikejima T (2008). Reactive oxygen species mediated oridonin-induced Hep G2 apoptosis through p53, MAPK, and mitochondrial signaling pathways. *Journal of Pharmacological Sciences*.

[21] Lee Y-J, Kuo H-C, Chu C-Y, Wang C-J, Lin W-C, Tseng T-H (2003). Involvement of tumor suppressor protein p53 and p38 MAPK in caffeic acid phenethyl ester-induced apoptosis of C6 glioma cells. *Biochemical Pharmacology*.

[22] Bai J, Cederbaum AI (2006). Cycloheximide protects HepG2 cells from serum withdrawal-induced apoptosis by decreasing p53 and phosphorylated p53 levels. *Journal of Pharmacology and Experimental Therapeutics*.

[23] Adams JM, Cory S (1998). The Bcl-2 protein family: arbiters of cell survival. *Science*.

[24] Fulda S, Meyer E, Debatin K-M (2000). Metabolic inhibitors sensitize for CD95 (APO-1/Fas)-induced apoptosis by down-regulating Fas-associated death domain-like interleukin 1-converting enzyme inhibitory protein expression. *Cancer Research*.

[25] Karpinich NO, Tafani M, Rothman RJ, Russo MA, Farber JL (2002). The course of etoposide-induced apoptosis from damage to DNA and p53 activation to mitochondrial release of cytochrome *c*. *The Journal of Biological Chemistry*.

[26] Borner C (2003). The Bcl-2 protein family: sensors and checkpoints for life-or-death decisions. *Molecular Immunology*.

[27] Komarov PG, Komarova EA, Kondratov RV (1999). A chemical inhibitor of p53 that protects mice from the side effects of cancer therapy. *Science*.

[28] Charlot JF, Nicolier M, Prétet JL, Mougin C (2006). Modulation of p53 transcriptional activity by PRIMA-1 and Pifithrin-*α* on staurosporine-induced apoptosis of wild-type and mutated p53 epithelial cells. *Apoptosis*.

[29] Kwon B-M, Lee S-H, Choi SU (1998). Synthesis and *in vitro* cytotoxicity of cinnamaldehydes to human solid tumor cells. *Archives of Pharmacal Research*.

[30] Wu S-J, Ng L-T, Lin C-C (2004). Effects of vitamin E on the cinnamaldehyde-induced apoptotic mechanism in human PLC/PRF/5 cells. *Clinical and Experimental Pharmacology and Physiology*.

[31] Wu S-J, Ng L-T, Lin C-C (2005). Cinnamaldehyde-induced apoptosis in human PLC/PRF/5 cells through activation of the proapoptotic Bcl-2 family proteins and MAPK pathway. *Life Sciences*.

[32] Cory S, Adams JM (2002). The BCL2 family: regulators of the cellular life-or-death switch. *Nature Reviews Cancer*.

[33] Yu W, Sanders BG, Kline K (2003). RRR-*α*-tocopheryl succinate-induced apoptosis of human breast cancer cells involves bax translocation to mitochondrial. *Cancer Research*.

[34] Ho P-J, Chou C-K, Kuo Y-H, Tu L-C, Yeh S-F (2007). Taiwanin A induced cell cycle arrest and p53-dependent apoptosis in human hepatocellular carcinoma HepG2 cells. *Life Sciences*.

[35] Gu H, Wang X, Rao S, Wang J, Zhao J, Ren FL (2008). Gambogic acid mediates apoptosis as a p53 inducer through down-regulation of mdm2 in wild-type p53-expressing cancer cells. *Molecular Cancer Therapeutics*.

[36] Komarova EA, Gudkov AV (2000). Suppression of p53: a new approach to overcome side effects of antitumor therapy. *Biochemistry*.

[37] Komarova EA, Gudkov AV (2001). Chemoprotection from p53-dependent apoptosis: potential clinical applications of the p53 inhibitors. *Biochemical Pharmacology*.

[38] Lorenzo E, Ruiz-Ruiz C, Quesada AJ (2002). Doxorubicin induces apoptosis and CD95 gene expression in human primary endothelial cells through a p53-dependent mechanism. *Journal of Biological Chemistry*.

